# Supplementary material for: Preeclampsia and Cerebral Palsy in Offspring
Source: Children (Basel). 2022 Mar 9;9(3):385. doi: 10.3390/children9030385 (PMC8947474; doi:10.3390/children9030385)
Supplement: Supplementary file 1 [file children-09-00385-s001.zip › children-1601039-supplementary.pdf]

Supplementary Materials

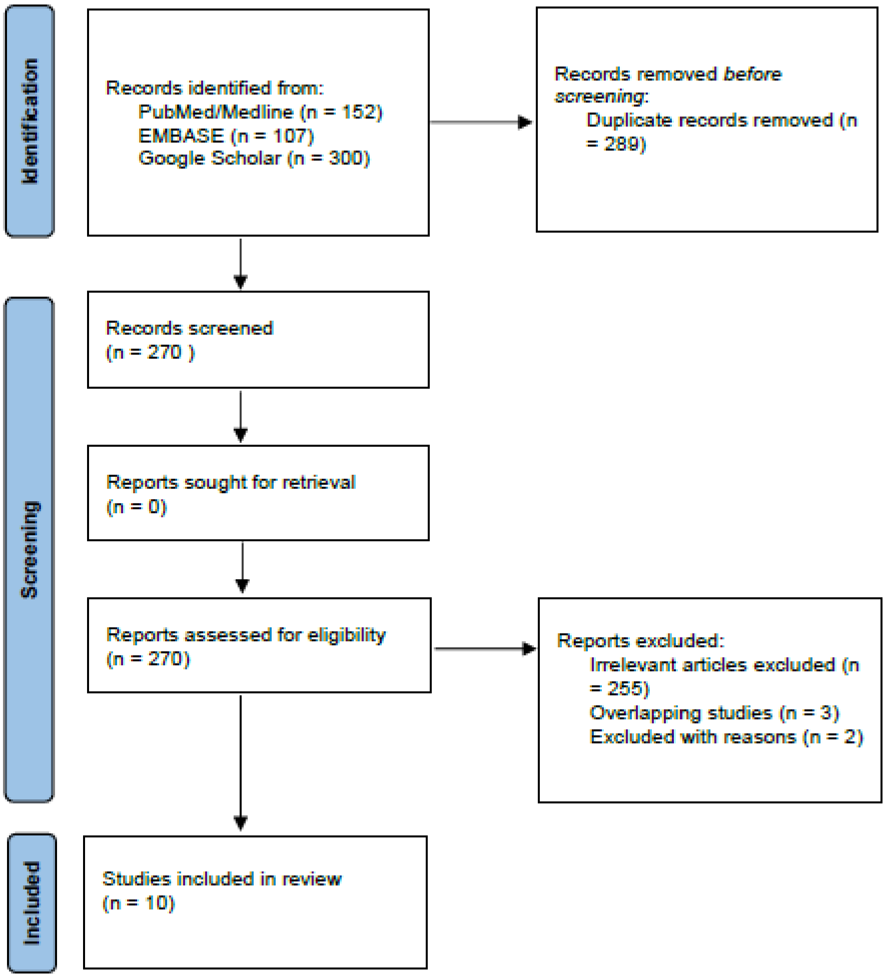

Figure S1. Flow chart presenting the steps in the selection of eligible studies.

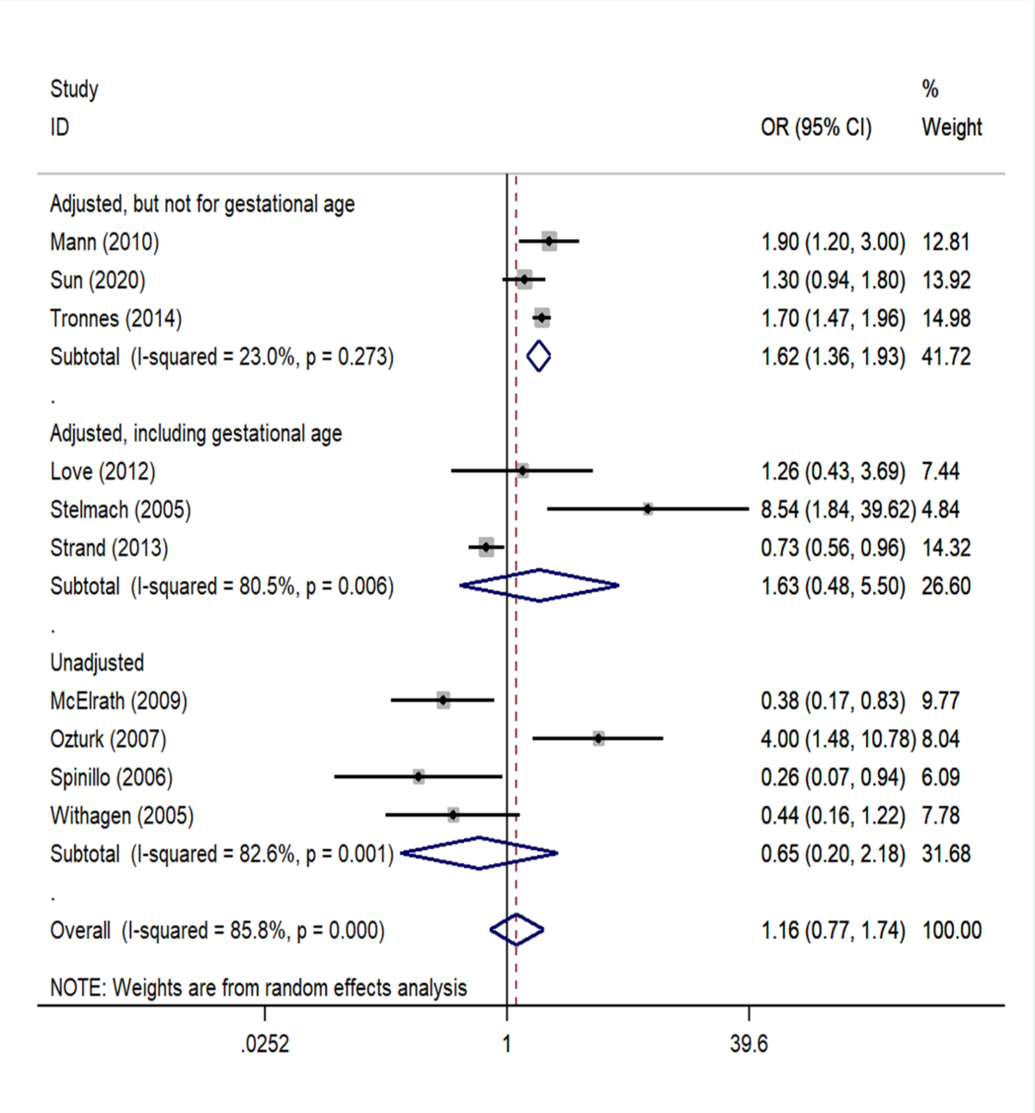

Figure S2. Forrest plot describing the association of preeclampsia with cerebral palsy and the subgroup analysis for studies provided adjusted odds ratios for several variables and gestational age, studies provided adjusted odds ratios for several variables, but not gestational age and studies provided unadjusted odds ratios.

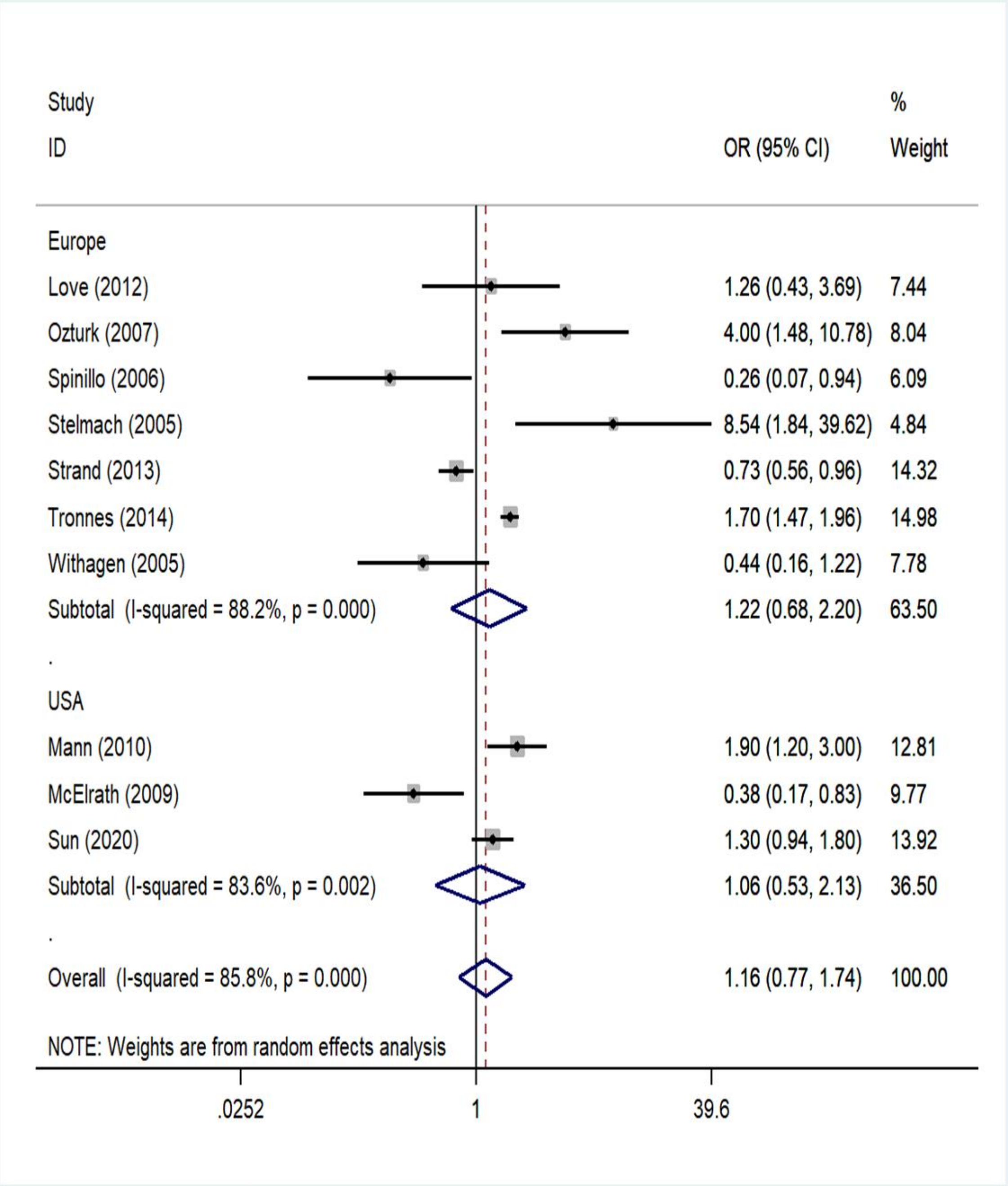

Figure S3. Forrest plot describing the association of preeclampsia with cerebral palsy based on the subgroup analysis for studies depending on geographical region.

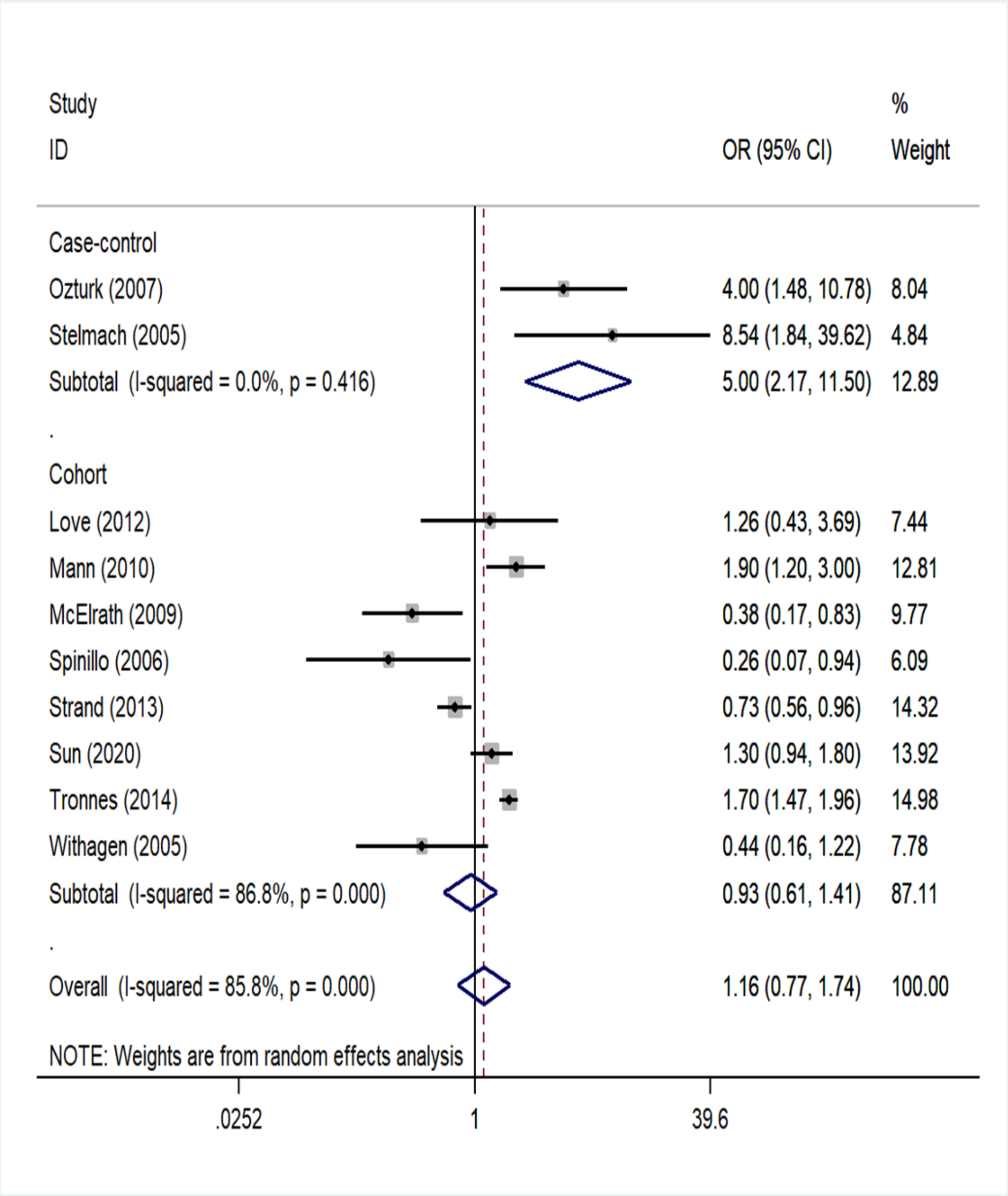

Figure S4. Forrest plot describing the association of preeclampsia with cerebral palsy based on the subgroup analysis for studies depending on study design.

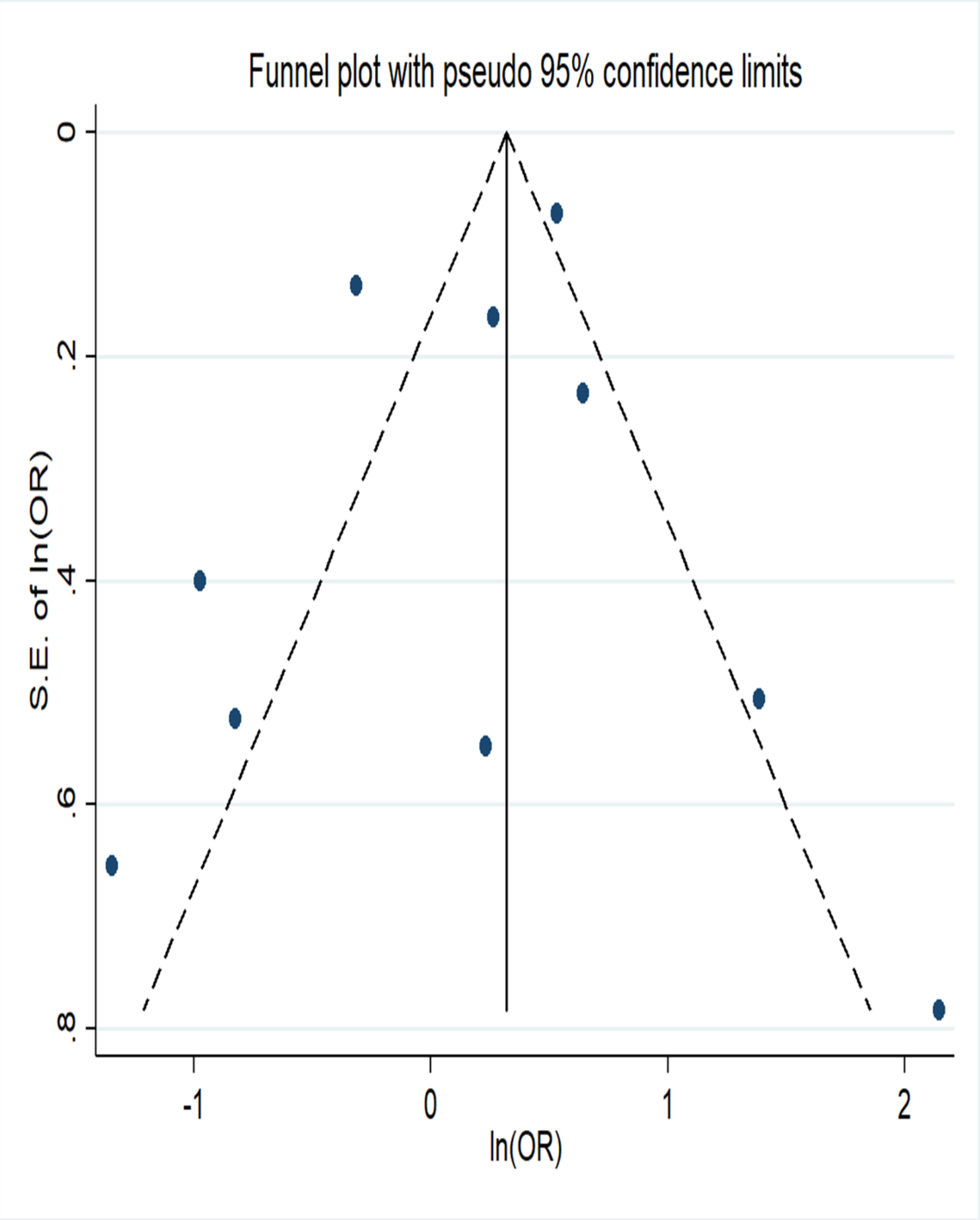

Figure S5. Funnel plot of the meta-analysis on association of preeclampsia with cerebral palsy showing evidence of publication bias.

Table S1. Studies excluded with their reason for exclusion.

Study	Title	Reason for exclusion
**Studies excluded due to overlap**		
Spinillo (1994)	Two-year infant neurodevelop-mental outcome after expectant	The study period 1986-1990 is overlapped from the study of Spinillo that took place on 2006 and its study period is 1983-2002. Also, the population was drawn from the same computer database

	management and indicated preterm delivery in hypertensive pregnancies	In Pavia, Italy and was extracted by taking into account the same characteristics
Spinillo (1998)	Preeclampsia, preterm delivery and infant cerebral palsy	The study period 1987-1993 is overlapped from the study of Spinillo that took place on 2006 and its study period is 1983-2002. Also, the population was drawn from the same computer database In Pavia, Italy and was extracted considering similar characteristics
Mor (2016)	Early onset preeclampsia and cerebral palsy: a double hit model?	The study period 1990-2013 is overlapped from the study of Nahum Sacks that took place on 2019 and its study period is 1991-2014. Also, the population was drawn from the same computer database In Soroka University Medical Center, Israel and was extracted considering similar characteristics
**Studies excluded due to other reasons**		
Blair (2016)	Cerebral palsy and perinatal mortality after pregnancy-induced hypertension across the gestational age spectrum: observations of a reconstructed total population cohort	The study included all cases of pregnancy-induced hypertension and preeclampsia and excluded due to authors' criteria
Sacks (2016)	Long-term neuropsychiatric morbidity in children exposed prenatally to preeclampsia	The study does not provide odds ratio for the association between preeclampsia and cerebral palsy

Table S2. Characteristics of all the eligible case-control studies

First author (Year)	Number of cases	Number of controls	Study design	Study period	Region	Definition/features of women with preeclampsia	Definition of infants with cerebral palsy	Main results
Stelmach (2005)	158	316	Case-Control	-	Tartu City, Estonia	153 cases and 268 controls (available for analysis)/ CP group: mean BW 2927.2gr, mean GA 36.1w/ Control group: mean BW 3494.3gr, GA varied from 30-43w	Several antenatal, intrapartum and neonatal factors associated with cerebral palsy examined	Out of 11 cases with preeclampsia 9 correlated with CP (81.8%) and out of 410 cases without preeclampsia 144 correlated with CP (35.1%%), by statistical software package R 1.6.2 - A Language and Environment/adjustment factors: sex, time of birth, place of residence
Ozturk (2005)	98	530	Case-Control	1990-2004	Duzce, Turkey	CP group: mean GA 38,1w, mean BW 2887gr, preeclampsia 7 cases / Control group: mean GA 39,2w, mean BW 3230, preeclampsia 10 cases	The diagnosis of CP was confirmed by at least two physicians (a pediatrician and a neurologist) /	Out of 17 cases with preeclampsia, 7 correlated with CP (41.2%), whereas among 611 cases without preeclampsia 91 correlated with CP/ statistics made with x2 test and Student's f-test / univariate analysis

Table S3. Characteristics of all the eligible cohort studies

First author (Year)	Cohort size	Incident cases	Follow-up period	Study design	Study period	Region	Definition/features of women with preeclampsia	Definition of infants with cerebral palsy	Main results
Withagen (2005)	574	21	Median 7 years	Cohort	-	Rotterdam, The Netherlands	Study group(192): mean GA 32w, mean BW 1215gr, Ventilation 101/ group I(192): mean GA 30w, mean BW 1337gr	Child morbidity and development were assessed by means of the	5/159 of mothers with preeclampsia developed CP (3.1%) and

							,Ventilation 160/ group II: mean GA 32w, mean BW 1785gr,Ventilation 103	Child Behaviour Checklist (CBCL) / The information obtained by means of the questionnaires was supplemented by review of medical records when available.	16/232 of mothers without preeclampsia developed CP (6.8%), p>0.05 by McNemar test / univariate analysis
Spinillo (2006)	534	50	2 years	Cohort	1983-2002	Pavia, Italy	Intact Survival: mean GA 30,2w, mean BW 1148gr, preeclampsia 120 cases/ Minor Abnormalities: mean GA 29,6w, mean BW 1131gr, preeclampsia 26 cases/ CP: mean GA 29w, mean BW 1049gr, preeclampsia 5 cases	Neurodevelopmental evaluation of the infants at discharge,3,6,9,12 and 24 months of corrected age	Out of 178 cases with preeclampsia 5 correlated with CP (2.8%) and out of 525 women without preeclampsia 45 correlated with CP(8.5%) (not referring to a test used) / univariate analysis
McElrath (2009)	1455	120	2 years	Cohort	2002-2004	Boston, USA	GA ranges from 23w to 27w of gestation with the majority at 27w(357 infants), BW ranges from <750gr(385 inf) to >1250gr(20 inf), Preeclampsia came up to 7 cases in the CP group	The neurologic evaluation was performed by examiners using a structured data collection form. /The topographic diagnosis of cerebral palsy (quadriparesis, diparesis, or hemiparesis) was based on an algorithm that used these data	Among 139 infants correlated with preeclampsia in mothers , 7 diagnosed with CP (5%), whereas among 917 infants without preeclampsia in mothers, 113 correlated with preeclampsia to mothers (12.3%), with Pearson's x2 or Fisher's exact test / univariate analysis
Mann (2010)	122.476	337	3 years	Cohort	1996-2002	South Carolina, USA	Singleton pregnancies and exclusion of pregnancies that performed before 21 weeks and have no opportunity for the presence for preeclampsia / Infants from singleton pregnancies and exclusion of infants with chromosome or genetic abnormality	Confirmed cases of children with CP according to ICD-9 / Diagnosis set by two different health care providers	Among 337 infants with CP, 22 correlated with preeclampsia to mothers (6.5%) and among 122.139 infants with CP, 4204 correlated with preeclampsia (3.4%), p=0.002 with X2 test or Fischer's test for categorical variables and Student's t-test for continuous variables /

									Models are adjusted for maternal age and race, genito-urinary infection in the first two trimesters and child's sex.
Love (2012)	28.967	-	-	Cohort	1995-2008	Aberdeen, UK	Women were divided in three categories depending on the hypertensive status / Out of 1774 women with preeclampsia 149 was very premature and 317 premature	Children recorded on the SNS were categorised according to their registered diagnoses. On investigation, there were over 95 different diagnoses documented so these were grouped into seven categories according to clinical judgement. These categories were 'congenital abnormality', 'cerebral palsy', 'autism', 'attention deficit hyperactivity disorder', 'developmental delay', 'communi- cation difficulties/learning difficulties' and 'other'	Preeclampsia as a maternal abnormality responsible for CP: Unadjusted OR 2,72 /Adjusted OR 1,26 (95% CI 0.43, 3.68)}, p=0,668 using the Statistical Package for the Social Sciences 17.0 (SPSS Inc. Chicago, Illinois) / All OR adjusted for maternal sociodemographic characteristics and simultaneously for other variables included in the model
Strand (2013)	617.506	849	-	Cohort	1996-2006	Trondheim, Norway	Women dichotomised as nulliparous and parous and depending on the time of birth as term, moderate preterm and very preterm / Children separated to SGA(=FGR) and defined SGA(<10th percentile)	Diagnosis of CP confirmed in all children when they were at least four years old, according to the recommendations of the Surveillance of Cerebral Palsy in Europe network	Among 22.956 mothers underwent preeclampsia in pregnancy , 75 correlated with CP (0.3%), whereas among 594.551 mothers without preeclampsia, 774 correlated with CP(0.13%), analyzing with x2 statistics and SPSS programme / adjusting factors: maternal age, parity, smoking in pregnancy, assisted fertilisation, and sex of the child and

									at the same time were checked for gestational age and small for gestational age
Tronnes (2014)	1.764.509	3151	>4 years	Cohort	1967-2001	Bergen, Norway	Exclusion criteria for the children was the week of gestation and the birth-weight in the corre-sponding gestational week (Children born <23w or >43w or with BW more than 3SDs ex-cluded)	CP cases were identified by the International Classification of Diseases codes 342.0 to 344.9 (9th revi-sion) and G80–G83.9 (10th re-vision) in the in-surance data-base	Out of the 50.209 cases with preeclampsia, 174 cases came up with CP [0.34%, crude OR 2.0 (1.7-2.3), ad-justed OR 1.7(1.5-2.0), whereas out of 1.714.300 cases without preeclampsia, 2977 cases came up with CP/ statistical analysis made by using PASW software, ver-sion 18.0; IBM SSPS Statis-tics, IBM Corp. NY, USA / ad-justed ORs for several factors, but not for ges-tational age
Sun (2020)	980.560	995	14 years	Cohort	1991-2009	Washing-ton, Seat-tle	Preeclampsia Group: mean GA 39.3w, mean BW 3463gr / Non-ex-posed to preeclampsia: mean GA 39.8w, mean BW 3628gr / Year of birth was included as a continuous variable - Maternal age was cate-gorized as 19 years or younger, 20 to 24 years, 25 to 29 years, 30 to 34 years, 35 to 39 years, or 40 years or older - Parity was dichotomized as primiparous or multipa-rous	A range of ad-verse neurode-velopmental outcomes—cerebral palsy, ADHD, ASD, epilepsy, intel-lectual disabil-ity, and vision and hearing loss— were ex-amined and the association be-tween expo-sure to preeclampsia and each out-come by lo-gistic regres-sion was inves-tigated	Among 28.068 mothers devel-oped preeclampsia during preg-nancy, 40 cor-related with cerebral palsy in the infants (0.14%), while among 952.492 mothers with-out preeclamp-sia, 965 corre-lated with CP in the infants (0.1%), in term singleton births/ Among all chil-dren term and preterm the corresponding percentages was 0.3% for the preeclamp-sia group and 0.1% for non-preeclamptic

								mothers / adjusted for sex, year of birth, mother’s age, parity, marital status, maternal and paternal educational levels, and parental immigrant status, but not for gestational age

Table S4. Evaluation of the eligible case-control studies with Newcastle-Ottawa scale

Study	Selection				Comparability	Exposure			Total
	Adequacy of case definition	Representativeness of cases	Selection of controls	Definition of controls	Comparability on age and other factors	Assessment of exposure	Same method of ascertainment for cases and controls	Non-response rate	
Stelmach (2005)	1	1	1	1	1	1	1	0	7
	Population-based prevalence study in the city and county of Tartu and were entered into a computer data- base.	One hundred fifty-eight cases of cerebral palsy of all severity stages (mild to severe) were initially ascertained / It is the biggest and most representative part of that region and belongs to the catchment area of the Children’s Clinic of Tartu University Clinics	Two controls for each case were selected from the whole population reg- ister of Tartu city and county in Estonia	The family physicians of control children were contacted by telephone to exclude possible unattended developmental problems in children with a minimum age of 4 years.	The controls were matched by sex, year and month of birth, and place of residence at the time of birth (urban or rural area), the latter being well descriptive of the socioeconomic status of families in the Estonian context.	Multiple sources of ascertainment were used to compile the database / o check the developmental status of control subjects, we searched through the hospital admission database and the register of out-patient visits to pediatric neurologists at Tartu University Clinics	Hospital admission database and the register of out-patient visits to pediatric neurologists at Tartu University Clinics.	Neither obstetric nor neonatal records could be found for 47 selected controls and 1 child with cerebral palsy. We excluded one child with cerebral palsy owing to missing data pertaining to the perinatal period	
Ozturk (2007)	1	1	1	0	2	1	0	0	6
	Cross-sectional study within the rural and urban area of Duzce province was performed between January 2006 and March 2006	All children with CP were sought from multiple sources, including the health services, population registry service, education authorities, the office	CP and to compare them with normal population, a cross-sectional study within the rural and urban area of Duzce	No description	Specific complications of pregnancy encompassing abortion, hemorrhage in late pregnancy, premature rupture of membranes (PROM), gestational diabetes, preeclamp-	All children with CP were sought from multiple sources, including the health services, population registry service, education authorities, the office of the village headman (Mukhtar) and associations for CP,	No	No description	

Study	Selection				Comparability	Exposure			Total
	Adequacy of case definition	Representativeness of cases	Selection of controls	Definition of controls	Comparability on age and other factors	Assessment of exposure	Same method of ascertainment for cases and controls	Non-response rate	
		of the village headman (Mukhtar) and associations for CP, and a rehabilitation center for disabled children.			sia, and preterm labor were examined.	and a rehabilitation center for disabled children / A structured questionnaire was filled it out face to face and mothers' past medical and obstetrics history were obtained			

Table S5. Evaluation of the eligible cohort studies with Newcastle-Ottawa scale

Study	Selection				Comparability	Outcome			Total
	Representativeness of the exposed	Selection of non-exposed	Ascertainment of exposure	Outcome not present at start	Comparability on age and other factors	Assessment of outcome	Long enough follow-up (median ≥2 years)	Adequacy (completeness) of follow-up (≥85% response rate)	
	1	1	1	0	2	1	1	0	7
Withagen (2005)	The study obtained approval of the Institutional Medical Ethics Committee. The study group consisted of all liveborn and one year surviving infants of 222 consecutive patients, admitted with severe pre-eclampsia between 24 and 32 weeks of gestation, who underwent haemodynamic treatment aimed at	Each infant of a pre-eclamptic mother was matched for gestational age, gender and year of birth in a blinded fash- ion, without knowledge of neonatal characteristics and clinical outcome, with two liveborn infants of non-pre- eclamptic mothers	The information obtained by means of the questionnaires was supplemented by review of medical records when available	No description	Gestational age, birthweight, umbilical arterial pH, ventilation, BPD, ICH, PVL	Questionnaires was supplemented by review of medical records	Yes	Follow-up rate <85% in all the three groups	

Study	Selection				Comparability	Outcome			Total
	Representativeness of the exposed	Selection of non-exposed	Ascertainment of exposure	Outcome not present at start	Comparability on age and other factors	Assessment of outcome	Long enough follow-up (median ≥2 years)	Adequacy (completeness) of follow-up (≥85% response rate)	
	prolongation of pregnancy								
	1	1	0	0	2	1	1	1	7
Spinillo (2006)	The study used a database containing obstetric and infant information of an historical cohort of all VLBW infants delivered at the Department of Obstetrics and Gynecology of the University of Pavia during the period 1983–2002.	All the VLBW infants born over a 20-year period (1983–2002) at a single institution.	No description	No description	Odds ratio and 95% CI as obtained by logistic regression equations containing neonatal death or cerebral palsy as a combined outcome variable, and gestational age, antenatal corticosteroid and postnatal surfactant use as explanatory variables.	Neurodevelopmental evaluation of the infants was carried out by a child neuropsychiatrist who was not involved in the intensive care of the infants. Examinations were carried out at discharge and after 3, 6, 12 and 24 months of corrected age. Neurological evaluation of the newborns was based on the methods of Amiel-Tison and Grenier.[12] The Bayley scales of infant development were used to assess cognitive development (mental developmental index [MDI] at 12 and 24 months).	Yes	534 (88.4%) completed the neurodevelopmental follow-up programme.	
	1	1	1	0	2	1	1	0	7
McElrath (2009)	During 2002–2004, women delivering before 28 weeks of gestation at one of 14 participating institutions	During 2002–2004, women delivering before 28 weeks of gestation at one of 14 participating institutions in 11 cities in 5 US	The neurologic evaluation was performed by examiners using a structured data collection form.	No description	Compared each of the pregnancy complications with pre- eclampsia and adjusted for gestational age and receipt of an antenatal steroid.	The neurologic evaluation was performed by examiners using a structured data collection form	Yes	No statement	

Study	Selection				Comparability	Outcome			Total
	**Representativeness of the exposed**	**Selection of non-exposed**	**Ascertainment of exposure**	**Outcome not present at start**	**Comparability on age and other factors**	**Assessment of outcome**	**Long enough follow-up (median ≥2 years)**	**Adequacy (completeness) of follow-up (≥85% response rate)**	
	in 11 cities in 5 US states were asked to enroll in the study. At each site, the enrollment and consent processes were approved by the local institutional review board.	states were asked to enroll in the study. At each site, the enrollment and consent processes were approved by the local institutional review board.							
	1	1	1	1	1	1	1	1	8
Mann (2010)	De-identified South Carolina Medicaid billing records for pregnancies and deliveries that occurred between 1996 and 2002 inclusive. We also obtained linked files to birth certificates and Medicaid billing records for children.	De-identified South Carolina Medicaid billing records for pregnancies and deliveries that occurred between 1996 and 2002 inclusive. We also obtained linked files to birth certificates and Medicaid billing records for children.	The outcome of CP was determined by identifying children diagnosed with CP in the Medicaid data	Therefore, we limited our analyses to 'confirmed cases' of CP, defined as children who were diagnosed with CP by at least two different health care providers	The regression models were adjusted for maternal age and race ('white', 'black' or 'other'), genitourinary infection occurring in the first two trimesters and child's sex / the primary models were estimated without controlling for gestational age	The outcome of CP was determined by identifying children diagnosed with CP in the Medicaid data	Yes	10 669 for whom the absence of CP could not be confirmed because they did not remain enrolled in Medicaid until at least age 3 and never enrolled in public schools;	
	1	1	1	1	2	1	0	0	7
Love (2012)	This was a retrospective cohort study and the population consisted of all children born to mothers in Aberdeen city and	This was a retrospective cohort study and the population consisted of all children born to mothers in Aberdeen city and dis-	The database from which the study population is derived - the Aberdeen Maternity and Neonatal Databank (AMND) has been in existence since 1950. The	The primary outcome of interest was whether a child had developed a record in the Support Needs System	All OR adjusted for maternal sociodemographic characteristics and simultaneously for other variables included in the model	The data stored in both the AMND and SNS databases are of high quality and consistency, with stringent coding criteria used by trained staff. They also	No reference	No statement	

Study	Selection				Comparability	Outcome			Total
	**Representativeness of the exposed**	**Selection of non-exposed**	**Ascertainment of exposure**	**Outcome not present at start**	**Comparability on age and other factors**	**Assessment of outcome**	**Long enough follow-up (median ≥2 years)**	**Adequacy (completeness) of follow-up (≥85% response rate)**	
	district between 1995 and 2008	trict between 1995 and 2008	Support Needs System (SNS) is part of a Scottish-wide database recording information about children who have additional support needs for more than six months and has been utilised in Grampian since 1998			claim a high degree of completeness. The data have been collected prospectively, eliminating recall bias			
	1	1	1	1	2	1	1	1	9
Strand (2013)	All singleton babies surviving the early neonatal period in Norway between 1 January 1996 and 31 December 2006 were eligible for this study	All singleton babies surviving the early neonatal period in Norway between 1 January 1996 and 31 December 2006 were eligible for this study	From the Medical Birth Registry of Norway we extracted data on pre-eclampsia in pregnancy, maternal health and delivery, and the early neonatal period.	1494 children born between 1996 and 2006 had a diagnosis of cerebral palsy. By 27 March 2012, detailed data for 381 of these children (25.5%) had not been recorded in the cerebral palsy registry. Since no information was available on these children, including birth dates, we were not able to exclude these children from the reference population	The regression models were adjusted for maternal age and race ('white', 'black' or 'other'), genitourinary infection occurring in the first two trimesters and child's sex / the primary models were estimated without controlling for gestational age	The recording of data in the cerebral palsy registry of Norway and linkage with the medical birth registry requires informed consent from the parents. In addition to this detailed consent based information, the habilitation centres report the total number of children with cerebral palsy for whom they care (summative information).	Yes	Small number lost	
	1	1	1	1	1	1	1	1	9
Tronnes (2014)	We identified all live births from 1967 to 2001 registered in the MBRN	We identified all live births from 1967 to 2001 registered in the MBRN	By using the personal identification number in an encrypted form, we linked information from the MBRN, Statistics Norway, and the	We also excluded children who died within the first year of life, since these children were not likely to have been diagnosed with CP	We calculated the odds ratios of CP according to gestational age and examined whether odds ratios changed after adjustment for sociodemographic fac- tors and year of birth,	CP cases were identified by the International Classi- fication of Diseases codes 342.0 to 344.9 (9th revision) and G80–G83.9	Yes	Small number lost	

Study	Selection				Comparability	Outcome			Total
	**Representativeness of the exposed**	**Selection of non-exposed**	**Ascertainment of exposure**	**Outcome not present at start**	**Comparability on age and other factors**	**Assessment of outcome**	**Long enough follow-up (median ≥2 years)**	**Adequacy (completeness) of follow-up (≥85% response rate)**	
			National Insurance Scheme. MBRN provided information on maternal health, pregnancy disorders, delivery, and birth		and after additional adjustment for pregnancy disorders / Not adjusted for gestational age	(10th revision) in the insurance database			
	1	1	1	1	1	1	1	1	8
Sun (2020)	We identified all singleton live births from January 1, 1991, to December 31, 2009, defining term births as children born at a gestational age of at least 37 completed weeks by ultrasonographic measure if available and otherwise by last menstrual period	We identified all singleton live births from January 1, 1991, to December 31, 2009, defining term births as children born at a gestational age of at least 37 completed weeks by ultrasonographic measure if available and otherwise by last menstrual period	Information on all pregnancies delivered in Norway after 16 weeks' gestation is reported to the Medical Birth Registry of Norway at delivery by the mother's clinician	Neurodevelopmental diagnoses of participants were obtained from *International Classification of Diseases, Ninth Revision*, and *International Statistical Classification of Diseases and Related Health Problems, Tenth Revision*, codes in the National Insurance Scheme registry	Multivariable logistic analyses for participant sex and year of birth, maternal age, parity, maternal marital status, maternal and paternal educational levels, and parental immigrant status. Year of birth was included as a continuous variable	Neurodevelopmental diagnoses of participants were obtained from *International Classification of Diseases, Ninth Revision*, and *International Statistical Classification of Diseases and Related Health Problems, Tenth Revision*, codes in the National Insurance Scheme registry / Data were linked across these population registries by each person's unique Norwegian identification number.	Yes	Small number lost	

Table S6. Results of the meta-analyses examining the association between pre-eclampsia during pregnancy and cerebral palsy in the offspring; subgroup analyses by degree of adjustment, study design, geographic region, overall quality rating

	n[§]	OR (95%CI)	Heterogeneity I^2, p
Overall analysis	10	1.16 (0.77-1.74)	85.8%, 0.000
Subgroups by degree of adjustment			
Adjusted, but not for gestational age	3	1.62 (1.36-1.93)	23.0%, 0.273
Adjusted, including gestational age	3	1.63 (0.48-5.50)	80.5%, 0.006
Unadjusted	4	0.65 (0.20-2.18)	82.6%, 0,001

Subgroups by study design			
Case-control studies	2	5.00 (2.17-11.50)	0.0%, 0.416
Cohort studies	8	0.93 (0.61-1.41)	86.8%, 0.000
Subgroups by geographic region			
Europe	7	1.22 (0.68-2.20)	88.2%, 0.000
USA	3	1.06 (0.53-2.13)	83.6%, 0.002
Subgroups by overall quality rating			
Low (NOS 1-3)		No studies	
Intermediate (NOS 4-6)	3	0.74 (0.14-3.92)	87.9%, 0.000
High (NOS 7-9)	7	1.30 (0.87-2.15)	85.2%, 0.000

§ number of studies.

Table S7. The Preferred Reporting Items for Systematic Reviews and Meta-Analyses (PRISMA) Checklist

Section and Topic	Item #	Checklist item	Location where item is reported
		**TITLE**	
Title	1	Identify the report as a systematic review.	p.1
		**ABSTRACT**	
Abstract	2	See the PRISMA 2020 for Abstracts checklist.	p.1
		**INTRODUCTION**	
Rationale	3	Describe the rationale for the review in the context of existing knowledge.	p. 1-3
Objectives	4	Provide an explicit statement of the objective(s) or question(s) the review addresses.	p.1
		**METHODS**	
Eligibility criteria	5	Specify the inclusion and exclusion criteria for the review and how studies were grouped for the syntheses.	p.3-4
Information sources	6	Specify all databases, registers, websites, organisations, reference lists and other sources searched or consulted to identify studies. Specify the date when each source was last searched or consulted.	p.3-4
Search strategy	7	Present the full search strategies for all databases, registers and websites, including any filters and limits used.	p.4,5
Selection process	8	Specify the methods used to decide whether a study met the inclusion criteria of the review, including how many reviewers screened each record and each report retrieved, whether they worked independently, and if applicable, details of automation tools used in the process.	p. 4, Table S1
Data collection process	9	Specify the methods used to collect data from reports, including how many reviewers collected data from each report, whether they worked independently, any processes for obtaining or confirming data from study investigators, and if applicable, details of automation tools used in the process.	p. 4, Table S1
Data items	10a	List and define all outcomes for which data were sought. Specify whether all results that were compatible with each outcome domain in each study were sought (e.g. for all measures, time points, analyses), and if not, the methods used to decide which results to collect.	p. 4
	10b	List and define all other variables for which data were sought (e.g. participant and intervention characteristics, funding sources). Describe any assumptions made about any missing or unclear information.	p. 4
Study risk of bias assessment	11	Specify the methods used to assess risk of bias in the included studies, including details of the tool(s) used, how many reviewers assessed each study and whether they worked independently, and if applicable, details of automation tools used in the process.	p. 4-5
Effect measures	12	Specify for each outcome the effect measure(s) (e.g. risk ratio, mean difference) used in the synthesis or presentation of results.	p. 4
Synthesis methods	13a	Describe the processes used to decide which studies were eligible for each synthesis (e.g. tabulating the study intervention characteristics and comparing against the planned groups for each synthesis (item #5)).	p. 4
	13b	Describe any methods required to prepare the data for presentation or synthesis, such as handling of missing summary statistics, or data conversions.	p. 4
	13c	Describe any methods used to tabulate or visually display results of individual studies and syntheses.	p. 4
	13d	Describe any methods used to synthesize results and provide a rationale for the choice(s). If meta-analysis was performed, describe the model(s), method(s) to identify the presence and extent of statistical heterogeneity, and software package(s) used.	p. 4
	13e	Describe any methods used to explore possible causes of heterogeneity among study results (e.g. subgroup analysis, meta-regression).	p. 4

Section and Topic	Item #	Checklist item	Location where item is reported
	13f	Describe any sensitivity analyses conducted to assess robustness of the synthesized results.	p. 4
Reporting bias assessment	14	Describe any methods used to assess risk of bias due to missing results in a synthesis (arising from reporting biases).	p. 4-5, Table S4 & S5
Certainty assessment	15	Describe any methods used to assess certainty (or confidence) in the body of evidence for an outcome.	-
**RESULTS**			
Study selection	16a	Describe the results of the search and selection process, from the number of records identified in the search to the number of studies included in the review, ideally using a flow diagram.	p. 5-6
	16b	Cite studies that might appear to meet the inclusion criteria, but which were excluded, and explain why they were excluded.	Table S1
Study characteristics	17	Cite each included study and present its characteristics.	Table S2 & S3
Risk of bias in studies	18	Present assessments of risk of bias for each included study.	Table S4 & S5
Results of individual studies	19	For all outcomes, present, for each study: (a) summary statistics for each group (where appropriate) and (b) an effect estimate and its precision (e.g. confidence/credible interval), ideally using structured tables or plots.	Figure S2,S3, S4, S5
Results of syntheses	20a	For each synthesis, briefly summarise the characteristics and risk of bias among contributing studies.	p. 5-6
	20b	Present results of all statistical syntheses conducted. If meta-analysis was done, present for each the summary estimate and its precision (e.g. confidence/credible interval) and measures of statistical heterogeneity. If comparing groups, describe the direction of the effect.	p.5-6 & Table S6
	20c	Present results of all investigations of possible causes of heterogeneity among study results.	p.6
	20d	Present results of all sensitivity analyses conducted to assess the robustness of the synthesized results.	p. 5-6
Reporting biases	21	Present assessments of risk of bias due to missing results (arising from reporting biases) for each synthesis assessed.	p.6
Certainty of evidence	22	Present assessments of certainty (or confidence) in the body of evidence for each outcome assessed.	p.6 & Table S4, S5
**DISCUSSION**			
Discussion	23a	Provide a general interpretation of the results in the context of other evidence.	p. 7-9
	23b	Discuss any limitations of the evidence included in the review.	p. 9
	23c	Discuss any limitations of the review processes used.	p. 9
	23d	Discuss implications of the results for practice, policy, and future research.	p. 9
**OTHER INFORMATION**			
Registration and protocol	24a	Provide registration information for the review, including register name and registration number, or state that the review was not registered.	ID: 306467
	24b	Indicate where the review protocol can be accessed, or state that a protocol was not prepared.	PROSPERO
	24c	Describe and explain any amendments to information provided at registration or in the protocol.	-
Support	25	Describe sources of financial or non-financial support for the review, and the role of the funders or sponsors in the review.	p. 9
Competing interests	26	Declare any competing interests of review authors.	p. 9
Availability of data, code and other materials	27	Report which of the following are publicly available and where they can be found: template data collection forms; data extracted from included studies; data used for all analyses; analytic code; any other materials used in the review.	p.9
